# *Pinus sylvestris* as a missing source of nitrous oxide and methane in boreal forest

**DOI:** 10.1038/srep23410

**Published:** 2016-03-21

**Authors:** Katerina Machacova, Jaana Bäck, Anni Vanhatalo, Elisa Halmeenmäki, Pasi Kolari, Ivan Mammarella, Jukka Pumpanen, Manuel Acosta, Otmar Urban, Mari Pihlatie

**Affiliations:** 1 Global Change Research Institute CAS, Bělidla 4a, CZ-603 00 Brno, Czech Republic; 2 Department of Physics, University of Helsinki, P.O. Box 48, FI-00014, Finland; 3 Department of Forest Sciences, University of Helsinki, P.O. Box 27, FI-00014, Finland; 4 Department of Environmental and Biological Sciences, University of Eastern Finland, P.O. Box 1627, FI-70211, Kuopio, Finland; 5 Department of Food and Environmental Sciences, University of Helsinki, P.O. Box 56, FI-00014, Finland

## Abstract

Boreal forests comprise 73% of the world’s coniferous forests. Based on forest floor measurements, they have been considered a significant natural sink of methane (CH_4_) and a natural source of nitrous oxide (N_2_O), both of which are important greenhouse gases. However, the role of trees, especially conifers, in ecosystem N_2_O and CH_4_ exchange is only poorly understood. We show for the first time that mature Scots pine (*Pinus sylvestris* L.) trees consistently emit N_2_O and CH_4_ from both stems and shoots. The shoot fluxes of N_2_O and CH_4_ exceeded the stem flux rates by 16 and 41 times, respectively. Moreover, higher stem N_2_O and CH_4_ fluxes were observed from wet than from dry areas of the forest. The N_2_O release from boreal pine forests may thus be underestimated and the uptake of CH_4_ may be overestimated when ecosystem flux calculations are based solely on forest floor measurements. The contribution of pine trees to the N_2_O and CH_4_ exchange of the boreal pine forest seems to increase considerably under high soil water content, thus highlighting the urgent need to include tree-emissions in greenhouse gas emission inventories.

Methane (CH_4_) and nitrous oxide (N_2_O) are naturally produced in soils. The net CH_4_ and N_2_O flux at the soil–atmosphere interface is a balance of gas production, consumption and transport processes within soil (Supplementary Fig. S1). CH_4_ is produced by anaerobic methanogenesis^1^ in water saturated soils and oxidized by methanotrophic bacteria^2^. N_2_O is mainly formed during denitrification, anaerobic dissimilatory nitrate reduction to ammonium, and aerobic nitrification^1^. Denitrification is the only process consuming N_2_O by reduction to N_2_.

In addition to gas diffusion at the soil surface and ebullition^1^, it has been shown that plant-mediated transport^3^,^4^,^5^,^6^,^7^,^8^,^9^,^10^,^11^,^12^,^13^ can contribute significantly to CH_4_ and N_2_O exchange between the pedosphere and the atmosphere (Supplementary Fig. S1). CH_4_ and N_2_O produced in the soil can be taken up by roots, diffuse across root cortex^3^,^6^, and be transported into the above-ground plant tissues. This transport occurs via intercellular spaces and the aerenchyma system^3^,^5^,^6^,^7^,^11^ and/or in xylem via the transpiration stream^4^,^5^,^8^,^13^. Release of CH_4_ and N_2_O into the atmosphere takes place via lenticels or stomata^3^,^6^,^11^,^13^. Both gases may also be formed in plants, either by microorganisms living within the plant^14^,^15^,^16^ or by physiological and photochemical processes^17^,^18^,^19^.

In recent decades, N_2_O and CH_4_ fluxes from plants have predominantly been investigated in herbaceous plants from wetlands. Studies in trees are rather rare and restricted mostly to stem flux measurements on wetland species. Particularly, those upland tree species lacking an aerenchyma system have been poorly investigated^8^,^9^,^13^,^20^. This is despite the fact that upland soils seem to be an important natural source of N_2_O^21^ and a strong natural sink of CH_4_^22^. Moreover, the current flux estimates of N_2_O and CH_4_ from forest ecosystems are based mostly on measurements from the forest floor, excluding the contribution of trees.

We quantified N_2_O and CH_4_ fluxes from stems, shoots (i.e. terminal branches of ca 15–20 cm length in upper canopy), and the forest floor of boreal forest dominated by Scots pine (*Pinus sylvestris* L.). We also investigated whether soil moisture level affects the N_2_O and CH_4_ exchange from trees and forest floor. This study is unique for its simultaneous determination of stem, shoot, and forest floor fluxes. Data were collected during May to July 2013 in a 50-year-old *P. sylvestris* stand^23^ in Southern Finland on two experimental plots (dimensions of 20 × 15 m, a distance of 100 m apart) with naturally differing soil volumetric water content (VWC): dry plot with 0.33 ± 0.030 m^3^ m^−3^, wet plot with 0.75 ± 0.016 m^3^ m^−3^ (mean ± standard error).

## Results and Discussion

### N_2_O fluxes

**Figure 1:**
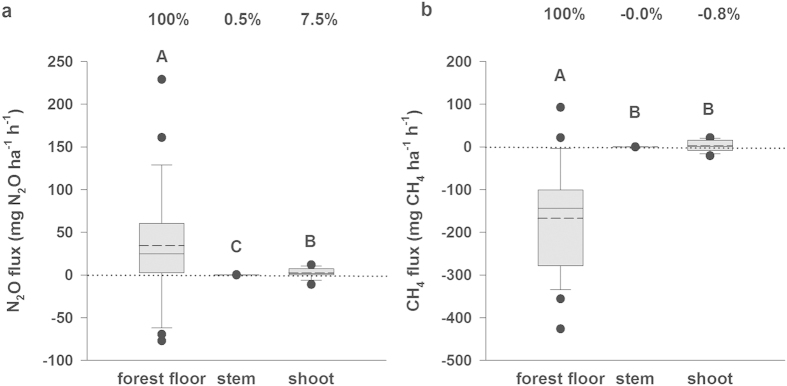
Scaled-up forest floor, stem and shoot fluxes of N_2_O (a) and CH_4_ (b) per unit ground area of dry boreal forest stand, dominated by Scots pine (*Pinus sylvestris*). Original flux rates per surface area of each ecosystem part are presented in Supplementary Fig. S2. Solid lines within the boxes mark medians, broken lines denote means, boundaries indicate 25th and 75th percentiles, and the whiskers 10th and 90th percentiles. Dots mark outliers. The plotted results are the medians/means of all sampling locations from the dry plot as follows: Forest floor fluxes are determined as medians and means of measurements from three soil chambers (n = 3) with nine measurement repetitions per chamber. Stem and shoot fluxes are expressed as medians and means of measurements on three trees (n = 3) with four to six repetitions per chamber. The fluxes from the shoots, stems and from the forest floor were measured simultaneously to allow their comparison. Contribution of stems and shoots to N_2_O and CH_4_ exchange are expressed as percentage of the forest floor flux. Statistically significant differences at *p* < 0.017 (multiple comparison – Bonferroni correction) between flux components are indicated by different capital letters above bars.

In dry field conditions typical for the studied boreal forest, we observed that *P. sylvestris* stems and shoots emitted N_2_O at rates (medians) of 0.023 and 0.097 μg N_2_O per m^2^ of stem and projected leaf area, respectively, per hour (Supplementary Fig. S2a), accounting for 0.11 and 1.9 mg N_2_O, respectively, after scaling up per hectare of ground area per hour (see Methods, Fig. 1a). To our knowledge, measurements of shoot fluxes of N_2_O from mature trees have never been reported, and most studies assume negligible shoot emissions compared to stem fluxes^5^,^9^,^10^,^12^,^13^. Contrary to this current understanding, the shoot fluxes of N_2_O from the studied pine trees exceeded the stem fluxes by more than 16 times. This underlines the important role of forest canopies in trace gas exchange. The N_2_O fluxes from pine trees were accompanied by forest floor flux rates reaching 2.50 μg N_2_O m^−2^ h^−1^ (24.9 mg N_2_O ha^−1^ h^−1^; Supplementary Fig. S2a, Fig. 1a), which agrees with previous soil N_2_O measurements in the same forest^24^. In general, boreal forest soils are characterized by low availability of mineral N^23^,^25^ and low N deposition^23^, resulting in low soil N_2_O emissions, particularly when compared to 4 to 12 times higher emissions from temperate and tropical forests^25^.

The up-scaled N_2_O emission rates from trees, assuming the mean tree constitution and density of 1000 trees per hectare in the dry plot (see Methods), were equivalent to 8.0% of the forest floor emissions per hectare of ground area (Fig. 1a, comparison of medians). Thus, the N_2_O emissions from trees constitute a significant part of the boreal pine forest N_2_O flux. The N_2_O flux from dry areas of the studied forest, including the contribution from forest floor and pine trees, reached approximately 26.9 mg N_2_O ha^−1^ h^−1^ (8.0 g CO_2_-e ha^−1^ h^−1^ using a global warming potential [GWP] of 298 [ref. 26]), which lies within the range of the global inventory estimates of N_2_O flux rates for boreal forests^25^. Based on the shoot-to-stem N_2_O fluxes ratio of 16 at the dry area, the shoot fluxes at the wet plot could reach 3.3 mg N_2_O ha^−1^ h^−1^ versus the measured stem fluxes of 0.20 mg N_2_O ha^−1^ h^−1^. As follows, under high soil water content typical for studied wet areas of the forest with density of 1400 trees per hectare, the contribution of pine trees could be up to 18% (based on medians comparison) of the forest floor N_2_O exchange.

Naturally, the up-scaled fluxes include uncertainties stemming from e.g. spatio-temporal variability in the fluxes, the use of mean stand density and constant shoot-to-stem flux ratio from the dry plot. The use of the constant shoot-to-stem flux ratio is justified based on the assumption that transport of N_2_O via the transpiration stream is the main driver for N_2_O emissions from the tree canopy^8^, and hence the stem emissions are directly reflected in the emissions from the canopy. Forest floor N_2_O and CH_4_ exchange is often characterized by high spatial variability, which has been also found to vary with distance to the trees^27^,^28^, while the variation in canopy N_2_O/CH_4_ exchange between individual pine trees as well as between different tree species remain unknown due to lack of canopy flux measurements. We estimated that temporal variability in the shoot, stem and forest floor N_2_O and CH_4_ fluxes was higher than the spatial variability in the dry plot, whereas in the wet plot the spatial variability dominated the fluxes. This indicates that most of the variability in the fluxes in the dominating dry areas originates from day-to-day variation, whereas the fluxes in the wet areas, which form a minority of the forest, are dominated by high small-scale variation.

The pine stem N_2_O fluxes correlated positively with forest floor fluxes (Spearman’s rank correlation coefficient: *ρ* = 0.351, *p* < 0.05), indicating that the tree-emitted N_2_O could originate from soil. As N_2_O is rather water soluble^4^, and many plant species emit N_2_O irrespective of the presence of an aerenchyma system^4^,^5^,^7^,^8^,^9^,^13^, we hypothesize that N_2_O is absorbed by roots from the soil, transported via xylem into the above-ground tree parts, and then emitted into the atmosphere.

### CH_4_ fluxes

Contrary to the CH_4_ uptake by shoots (i.e. negative flux) found in Scots pine seedlings grown under field and laboratory conditions^20^, we observed emissions of CH_4_ from both shoots and stems of mature *P. sylvestris*. This difference in shoot CH_4_ fluxes may result from (i) different soil water content and soil temperature (not reported for the seedlings experiment^20^), (ii) known discrepancy in emission capacities of young and mature trees^12^, and (iii) the fact that the seedlings were investigated in the absence of UV radiation^20^, which is known to stimulate CH_4_ formation^17^,^18^. The CH_4_ emission rates from pine stems and shoots were 0.005 and 0.050 μg CH_4_ m^−2^ h^−1^ (medians), respectively (Supplementary Fig. S2b). Up- scaled emission rates at stand level were 0.03 and 1.1 mg CH_4_ ha^−1^ h^−1^ (Fig. 1b) assuming mean tree constitution and density of 1000 trees per hectare (see Methods). As is the case of N_2_O, pine shoots seem to be the primary tree surface emitting CH_4_ into the atmosphere, given that shoot fluxes were 41 times higher than the stem fluxes. This contradicts the common assumption^5^,^9^,^10^,^11^ that basal regions of stems are the main source of CH_4_ and N_2_O from trees.

Whereas trees were a source of CH_4_, the forest floor was a sink (−14.4 μg CH_4_ m^−2^ h^−1^, Supplementary Fig. S2b; −143 mg CH_4_ ha^−1^ h^−1^, Fig. 1b). The estimated average pine tree CH_4_ emission represented 0.8% of the forest floor uptake. The CH_4_ uptake from the dry area of the studied forest (−4.9 g CO_2_-e ha^−1^ h^−1^ using GWP of 34 [ref. 26]) is roughly 35% to 50% lower than are estimates of CH_4_ uptake for boreal forests in global inventories^25^,^29^.

**Figure 2:**
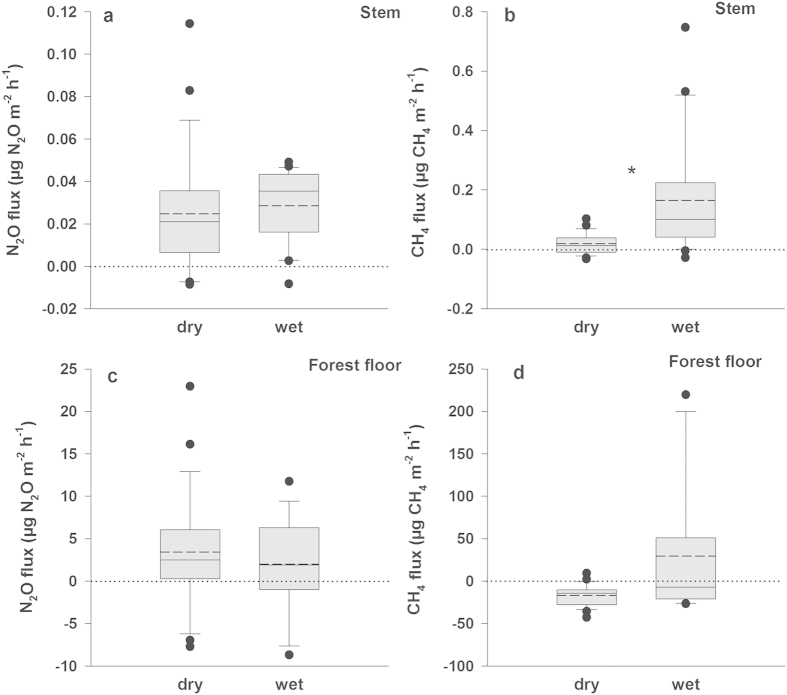
Stem and forest floor fluxes of N_2_O (**a,c**) and CH_4_ (**b,d**) from dry and wet plots of boreal forest dominated by Scots pine (*Pinus sylvestris*). Mean volumetric water contents for dry and wet plots (±s.e.) were 0.33 ± 0.030 m^3^ m^−3^ and 0.75 ± 0.016 m^3^ m^−3^, respectively. Flux rates of all sampling points as follows are expressed as medians (solid lines) or means (broken lines) per m^2^ of surface area. Stem fluxes are determined from six trees per plot (n = 6; 3–6 measurement repetitions per tree), and forest floor fluxes from three soil chambers per plot (n = 3; 6–9 repetitions per chamber). The fluxes from the stems and from the forest floor were always measured simultaneously. Statistically significant differences at *p* < 0.05 are indicated by an asterisk. For box plots description, see Fig. 1.

The median stem CH_4_ fluxes at the wet plot (0.100 μg CH_4_ m^−2^ h^−1^) were one order of magnitude higher than those at the dry plot (0.013 μg CH_4_ m^−2^ h^−1^) (Fig. 2b), while the soil remained a sink for CH_4_ even under high soil VWC (−7.09 μg CH_4_ m^−2^ h^−1^, −70.7 mg CH_4_ ha^−1^ h^−1^; Fig. 2d). Moreover, the stem-to-forest-floor CH_4_ fluxes ratio increased with soil VWC, underlining the importance of pine trees at wet areas in the balance of CH_4_. Although direct measurement of shoot CH_4_ flux at the wet plot was technically impossible, based on the shoot-to-stem CH_4_ fluxes ratio of 41 at the dry plot, the shoot CH_4_ fluxes at the wet plot were estimated to reach 24 mg CH_4_ ha^−1^ h^−1^ in comparison to the stem CH_4_ fluxes of 0.59 mg CH_4_ ha^−1^ h^−1^. Under high soil VWC and stand density of 1400 trees per hectare, CH_4_ emissions from pine trees could, therefore, account for up to 35% of the forest floor uptake. This estimate is rather higher than in a recent study by Pangala and colleagues, who found that CH_4_ emissions mediated by *Alnus glutinosa* and *Betula pubescens* contribute up to 14% to the total CH_4_ fluxes from a temperate forested wetland^12^.

The partial soil origin of pine-emitted CH_4_ is supported by strong positive correlation of stem CH_4_ fluxes with forest floor CH_4_ fluxes (*ρ* = 0.716, *p* < 0.001) and VWC in topsoil (*ρ* = 0.802, *p* < 0.001). In wet conditions, pine trees may therefore prevent CH_4_ consumption in the upper soil layers by transporting CH_4_, produced in deeper soil, into the atmosphere. We suggest that soil-produced CH_4_ is transported into the above-ground parts of *P. sylvestris* mainly by the transpiration stream and then released into the atmosphere predominantly via stomata^4^,^5^,^8^,^9^, thus explaining the higher CH_4_ emissions from shoots as compared to stems. This assumption is supported by the positive correlation between the shoot CH_4_ flux and transpiration (*ρ* = 0.626, *p* < 0.05), and stem CH_4_ flux and sap flow (*ρ* = 0.390, *p* < 0.01). Therefore, alternative pathways, such as radial diffusion of CH_4_ (and N_2_O) in stems through intercellular spaces of the ray parenchyma and a release from the stem via lenticels^11^,^30^,^31^, seem of a lesser importance.

Different mechanisms of CH_4_ emissions from trees grown on dry plot as compared to those on wet plot are, however, likely. Limited soil CH_4_ production in deeper mineral soil layers^32^ and low mineral soil VWC (0.28 ± 0.02 m^3^ m^−3^) in the studied period give an assumption of negligible soil CH_4_ production in the dry plot. Moreover, approximately half of the root system of *P. sylvestris* is located in the top soil organic layer with the rest of the roots equally distributed to mineral soil (0–40 cm)^33^. Therefore, it is probable that part of the CH_4_ emitted from trees in the dry plot originated from anaerobic production processes within the wood^14^,^15^,^16^ and/or aerobic, non-microbial metabolic processes in the plant tissues^17^,^18^.

*P. sylvestris* appears to be one of the missing sources for N_2_O and CH_4_ in boreal forests. N_2_O emissions from boreal pine forests may previously have been underestimated and the uptake of CH_4_ overestimated. Even though our measurements indicate only potential mechanisms, and more detailed measurements of spatio-temporal variability are necessary, the pine mediated N_2_O and CH_4_ emissions could account for up to 18% of forest floor N_2_O emissions and 35% of forest floor CH_4_ uptake, respectively, under high soil moisture conditions. This can be crucial for the future greenhouse gas budgets of boreal pine forests, especially if precipitation and evapotranspiration patterns will change due to climate change. Our findings highlight the important, but often neglected role of upland trees in N_2_O and CH_4_ exchange between the biosphere and the atmosphere and the importance of including tree emissions to the total forest ecosystem budgets of N_2_O and CH_4_.

## Methods

### Site description and experimental design

The measurements were performed in a 50-year-old stand of Scots pine (*Pinus sylvestris* L.) at the SMEAR II station (Station for Measuring Ecosystem–Atmosphere Relations) in Hyytiälä, Southern Finland (61°51′N, 24°17′E, 181 m a.s.l.) from 23 May to 19 July 2013. Established in 1962, the site is a boreal coniferous forest dominated by *P. sylvestris* with some additional Norway spruce (*Picea abies*) and broadleaved trees in the understorey^23^,^34^. The long-term annual mean temperature and precipitation are 3.5 °C and 711 mm, respectively^35^. The soil is Haplic podzol on glacial till with irregularly distributed peat soil spots^36^.

Naturally wet and dry plots (dimensions of 20 × 15 m, a distance of 100 m apart) with mean soil volumetric water content (VWC) 0.75 ± 0.016 m^3^ m^−3^ (mean ± standard error) and 0.33 ± 0.030 m^3^ m^−3^, respectively, were selected. During the measurement period, soil water content was measured using an HH2 Moisture Meter and Theta Probe (type ML2x, AT Delta-T Devices, Cambridge, UK) in A-horizon corresponding to depths 0–5 cm from the soil surface, and expressed as mean of three independent measurements close to each tree and soil chamber. Soil temperature was measured continuously by a DS1921G Maxim Thermochron iButtons (Maxim Integrated, San Jose, California, USA) in A-horizon next to each soil chamber.

On each plot, six representative trees were chosen for stem flux measurements (n = 6). Shoot fluxes were measured from the upper canopy of three trees used for the stem flux measurements at the dry plot. Shoot fluxes were not measured from the wet plot as installation of a scaffold tower was technically not possible. Forest floor CH_4_ and N_2_O fluxes were measured at three representative positions in the dry plot and at three positions in the wet plot (n = 3).

Four flux measurement campaigns, each taken over ca 2 weeks, were made for stem, shoot, and forest floor fluxes between 23 May and 19 July 2013 (for details concerning the number of replicates see the legends of Figs 1 and 2, and Supplementary Fig. S2 describing individual measuring campaigns). Simultaneous measurements of fluxes from each tree and the forest floor in its vicinity allowed a comparison of N_2_O and CH_4_ fluxes between tree shoots, stems, and forest floor. The fluxes were determined on all measuring days at approximately same time to prevent possible variation in flux rates caused by flux diurnal cycle.

The mean tree height, length of living crown, and stem diameter at breast height (DBH) of the selected pine trees were 18.2 ± 0.4 m, 6.47 ± 0.32 m, and 0.162 ± 0.012 m, respectively, for the wet plot. For the dry plot, these were 17.7 ± 0.5 m, 7.22 ± 0.43 m, and 0.180 ± 0.004 m, respectively. These morphological parameters did not differ significantly when comparing wet and dry plots. The stand densities were estimated to be 1000 and 1400 trees per hectare on the dry and wet plots, respectively. The stand basal area was measured directly on the plots using a rod relascope technique and was 19.5 and 26 m^2^ ha^−1^ on dry and wet plots, respectively.

### Chamber systems

The stem fluxes were measured using 12 stem chambers (1 chamber per tree) enclosing the entire stem circumference^37^^-*modified*^. The skeleton of the stem chamber (volume between 0.0009 and 0.0015 m^3^ depending on stem diameter) was created by a flexible pipe from polyethylene-coated aluminium (Synflex, Eaton Hydraulics Group Europe, Morges, Switzerland), which was wrapped in a spiral around the stem. A tube-fitting brace was attached to this spiral and enabled fixation of inlet and outlet connectors. Teflon FEP film (0.05 mm thick, Fluorplast, Maalahti, Finland) impermeable for CH_4_ and N_2_O was wrapped 1.5 to 2 times around the tube spiral to create the chamber wall, and then sealed with adhesive FEP tape. Due to the requirement of mounting the stem chambers on the basal part of the rough pine bark (around 0.2 m above the forest floor), the surface of the dead outer bark was carefully removed from the upper and basal ends of the chamber. The upper and basal ends of the Teflon foil were sealed with elastic closed cell polyethylene foam and wide flexible ties to the carefully smoothed bark surface. The results of the stem flux measurements on twelve trees were used in the comparison of the stem and forest floor fluxes between dry and wet plots (Fig. 2).

Two different shoot chamber types were used to measure fluxes of CH_4_ and N_2_O: two cylindrical chambers with FEP foil walls^38^ (volume 0.0054 m^3^) and a methacrylic cylindrical shoot chamber^39^ (volume 0.005 m^3^). We did not observe any differences in flux rates obtained by these two types of chambers. The three chambers were installed in the upper canopy of the three trees on the dry plot. The air temperatures (DT 612 thermometer, CEM, Shenzhen, China) inside and outside of the chambers were regularly measured during chamber closures. To avoid overheating in the chambers, the shoot fluxes were measured only on cloudy days. The comparison of the shoot, stem and forest floor fluxes presented in Fig. 1 and Supplementary Fig. S2 is based on the measurements at the dry plot only. To compare the whole tree flux rates in dry and wet plots, we used the shoot-to-stem flux ratio from the dry plot where both shoot and stem flux measurements were performed.

In both stem and shoot chambers, the mixing of the air inside the chambers was provided by vacuum pumps (V 1500-GAS-12V standard vacuum pumps, Xavitech, Härnösand, Sweden; NMP 850.1.2. KNDC B, KNF Neuberger, Freiburg, Germany) gas-tightly connected to the chamber using Teflon tubes and stainless steel connectors (Swagelok, Ohio, USA). The chambers were non-steady-state flow-through chambers returning the air from the pump again into the chambers. Gas samples were taken with a syringe via a septum connected to the air circulation. Six gas samples (each 20 ml) were taken from the closed stem and shoot chambers at time intervals of ca 60 min over a period of 6 h. The possible under-pressure resulted from the gas sampling was compensated by the flexible foil wall. The stem chambers were flushed with ambient air for at least 30 min before sampling.

Forest floor CH_4_ and N_2_O fluxes were measured using large opaque soil chambers made of aluminium^40^^- chamber #13^. Three chambers were placed on the dry plot (volume of ca 0.091 m^3^ depending on vegetation inside the chamber, enclosed soil surface area of 0.298 m^2^), and three on the wet plot (volume of ca 0.133 m^3^, soil area of 0.298 m^2^). The chambers were located in the vicinity of the measured trees. The ground vegetation in the soil chambers varied among chambers depending on the soil conditions and location, and consisted of *Sphagnum* sp., *Polytrichum* sp., *Dicranum polysetum*, *Pleurozium schreberi*, *Equisetum sylvaticum*, *Vaccinium myrtillus*, *Vaccinium vitis-idaea*, *Trientalis europaea*, and several representatives of *Poaceae*. The placement of chamber collars took place several days before the first sampling to allow the soil to settle and avoid soil disturbances. The soil chambers were closed for ca 40 min during which gas samples (each 20 ml) were taken at 2, 5, 10, 20, 30, and 40 minutes after the chamber closure. A fan was used to mix the headspace air during the closure. Chamber headspace temperature (DT 612 thermometer, CEM, China) was regularly monitored during the measurements.

### Gas analyses

Gas samples from stem, shoot, and soil chambers were taken in 20 ml syringes (BD syringe, Franklin Lakes, New Jersey, USA) and immediately transferred to the evacuated 12 ml glass vials (Labco, Ceredigion, UK), then stored at 4 °C. The gas samples were analysed by an Agilent 7890A gas chromatograph (GC) (Agilent Technologies, Santa Clara, California, USA) equipped with a flame ionization detector (FID) and an electron capture detector (ECD) for CH_4_ and N_2_O analyses, respectively^40^. Briefly, CH_4_ was detected by FID (300 °C) supplied with synthetic air (450 ml min^−1^) and hydrogen (H_2_, 45 ml min^−1^) and with nitrogen (N_2_, 5 ml min^−1^) as a make-up gas. N_2_O was detected using the ECD (380 °C) supplied with argon/methane (15 ml min^−1^) as a make-up gas. Helium (He, 45 ml min^−1^) was used in both cases as a carrier gas. Columns Porapak Q 80–100 Mesh and Hayesep Q 80–100 Mesh (Agilent Technologies, USA) were used for water vapour removal and gas separation. Oven temperature was kept at 60 °C. Retention times for CH_4_ and N_2_O were 3.6 and 4.3 min, respectively. The gas samples were automatically injected by an autosampler Gilson GX-271 Liquid Handler (Gilson, Middleton, Wisconsin, USA). An overpressure in vials was necessary for proper injection of gas samples and was an indicator of gas tightness of the vials. ChemStation B.03.02 software was used for the GC analyses.

The identification of CH_4_ and N_2_O peaks in gas samples and calculation of their molar fractions referred to dry air (hereinafter: “concentrations”) were performed using a four-point standard curve with the following concentrations: CH_4_ (1.207, 1.810, 2.413, 3.017 ppm of CH_4_ in synthetic air), N_2_O (0.279, 0.330, 0.381, 0.457 ppm of N_2_O in synthetic air). The four standards were analysed at the beginning of the analyses and after every ca 30 gas samples. A running standard (1.810 ppm CH_4_, 0.330 ppm N_2_O; in synthetic air) for detailed control was applied after every ca 15 gas samples.

### Calculation of N_2_O and CH_4_ flux rates

The flux rates of N_2_O and CH_4_ from stems, shoots, and forest floor were calculated by linear least square fits of time series of N_2_O and CH_4_ concentrations as follows


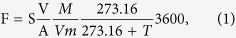


where *F* is flux of N_2_O or CH_4_ from stem, shoot, or forest floor surface [μg m^−2^ (surface area) h^−1^]; *S* is the slope of the linear fit to the N_2_O or CH_4_ concentrations over the chamber closure (ppm s^−1^); *V* is volume of chamber [m^3^]; *A* is stem surface area, projected leaf area, or soil surface area enclosed in stem, shoot, or soil chamber, respectively [m^2^]; *M* is molecular mass of N_2_O or CH_4_ [44.01 and 16.04 g mol^−1^, respectively]; *V*_*m*_ is molar volume of an ideal gas at 1 atmosphere pressure and 25 °C [0.0245 m^3^ mol^−1^]; and *T* the temperature [°C] inside the chamber. The stem surface area was estimated as a smooth cylinder around the bark because the micro-topography of the bark (very rough surface) makes any other methods ambiguous. The projected leaf area of shoots enclosed in the shoot chambers was determined by applying a destructive method at the end of the measurement campaign using an LI-3000 portable area meter (Li-Cor, Lincoln, Nebraska, USA).

The flux rates of N_2_O and CH_4_ were further estimated for the entire stem and projected needle area of each tree using the following parameters: The stem surface area (3.6–6.2 m^2^ per tree) was calculated as the lateral surface area of a right circular cone using the stem diameter at breast height (DBH) and the tree height. The needle biomass was determined using an allometric biomass equation (based on DBH, tree height, and length of living crown) for Scots pine^41^^-equation n. 27^ and used to calculate the entire projected needle area of each tree (10–31 m^2^ per tree) by multiplying the biomass weight with specific leaf area determined for *P. sylvestris* at the SMEAR II station^42^. The CH_4_ and N_2_O fluxes from stems, shoots, and forest floor were scaled up to 1 hectare of 50-year-old boreal pine stand using the estimated forest density and stand basal area (see chapter “Site description”).

The flux estimates and upscaling to a stand level are saddled with uncertainties arising from sampling and gas analyses^43^, variables in Equation 1, and application of allometric relationships for estimation of total leaf area^41^ and from calculation of stem area per tree. At a stand level, uncertainties of flux rates are thus particularly given by spatio-temporal variability in the fluxes, heterogeneity of tree morphological parameters (height, length of living crown, stem diameter), and stand heterogeneity (tree density per hectare and species composition etc.). In addition, fast changes in transpiration and sap flow rates induced by dynamic light environment under variable sky conditions have also a potential to substantially influence the gas exchange over longer periods^44^. However, here such factors are of minor importance, as the measurements were predominantly conducted during overcast days.

### Ancillary measurements

The following continuously measured variables at the SMEAR II experimental station were used for correlation analyses: a) soil water content (TDR-100, Campbell Scientific, North Logan, Utah, USA)^45^,^46^ and b) soil temperature (Philips KTY81, NXP, Eindhoven, Netherlands)^46^, both in four soil horizons (O-, A-, B- and C-horizon; corresponding to depths of −4–0, 0–5, 5–23 and 23–60 cm from the mineral soil surface); c) air temperature at 4.2 m height within the forest stand (Pt100 sensors), d) photosynthetic photon flux density at 23 m height (Li-190SZ, Li-Cor, USA); on *P. sylvestris*: e) stem sap flow using the Granier-type heat dissipation method^47^,^48^ at a height of about 2 m; and f) shoot transpiration at the top canopy with dynamic enclosures^49^.

### Statistics

Datasets were tested for normal distribution (Shapiro–Wilk test) and homogeneity of variances in different subpopulations. The flux data were assumed independent. Because of non-normally distributed data and/or data with unequal variances, the non-parametric Mann–Whitney rank sum test was run at *p* < 0.05 to test the statistical significance a) among flux rates from stems, shoots, and forest floor; and b) between flux rates from dry and wet plots.

Correlation analyses (a) between stem, shoot, and forest floor flux rates of N_2_O or CH_4_, and (b) between the trace gases flux rates and micro-climatic and other tree parameters were performed using non-parametric correlation analyses (Spearman’s rank correlation). The statistical significance was defined at *p* < 0.05.

SigmaPlot 11.0 (Systat Software, San Jose, California, USA) was used for statistical analyses.

## Additional Information

**How to cite this article**: Machacova, K. *et al. Pinus sylvestris* as a missing source of nitrous oxide and methane in boreal forest. *Sci. Rep.* **6**, 23410; doi: 10.1038/srep23410 (2016).

## Supplementary Material

Supplementary Information
