# Supplementary material for: Pinus sylvestris as a missing source of nitrous oxide and methane in boreal forest
Source: Sci Rep. 2016 Mar 21;6:23410. doi: 10.1038/srep23410 (PMC4800674; doi:10.1038/srep23410)
Supplement: Supplementary Information [file srep23410-s1.pdf]

Supplementary information

Pinus sylvestris as a missing source of nitrous oxide and methane in boreal forest

Katerina Machacova[1], Jaana Bäck[2,3], Anni Vanhatalo[3], Elisa Halmeenmäki[2], Pasi Kolari[2,3], Ivan Mammarella[2], Jukka Pumpanen[4], Manuel Acosta[1], Otmar Urban[1] & Mari Pihlatie[2,5]

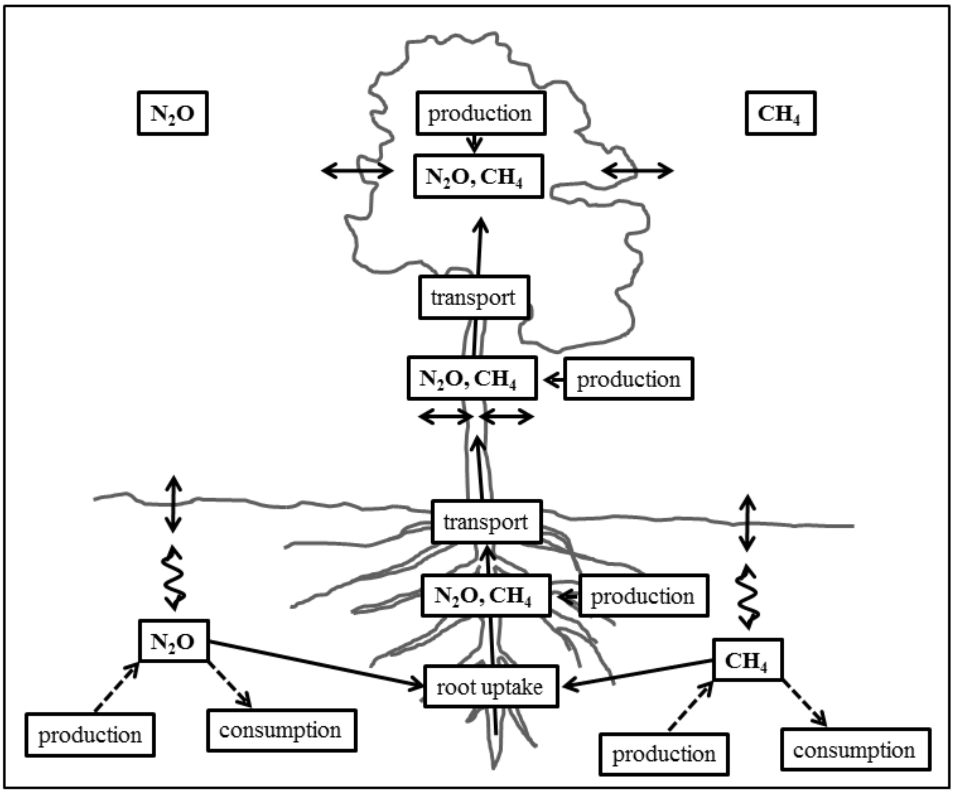

Supplementary Figure S1. Schematic illustration of N_2O and CH_4 fluxes in soil-tree-atmosphere continuum. Emission (i.e. positive flux) – gas flux from the soil and/or tree into the atmosphere, deposition (i.e. uptake, negative flux) – gas flux from the atmosphere into the soil and/or tree. For description, see the introduction text.

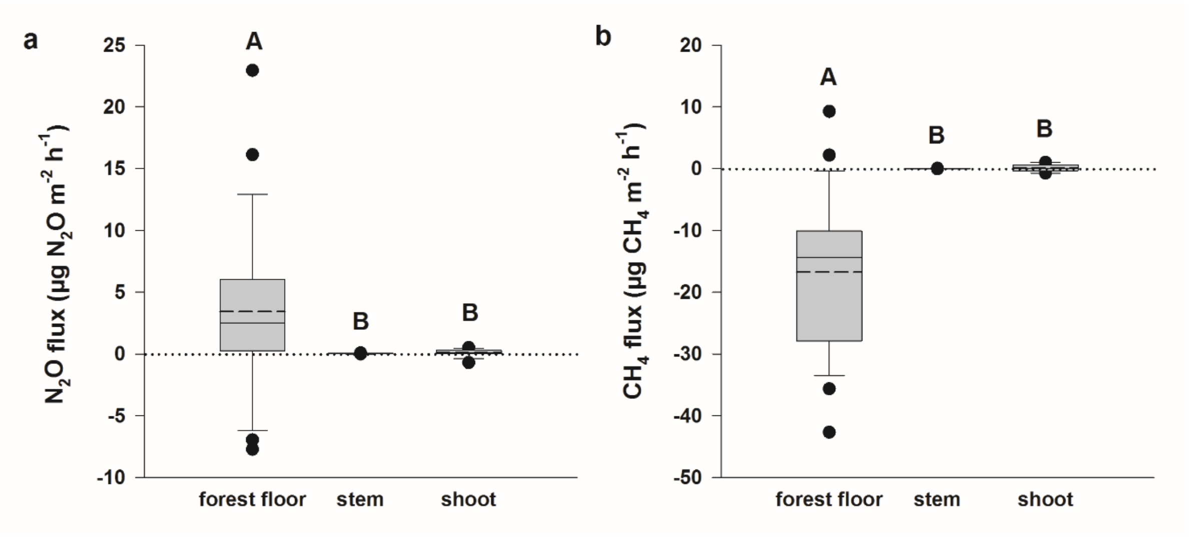

Supplementary Figure S2. Forest floor, stem and shoot fluxes of N_2O (a) and CH_4 (b) measured in dry boreal Scots pine (*Pinus sylvestris*) forest and expressed per unit area of each ecosystem part. The up-scaled ecosystem data per unit ground area are presented in Fig. 1. Solid lines within the boxes mark medians, broken lines denote means. Boundaries of the boxes indicate 25th and 75th percentiles, whiskers 10th and 90th percentiles. Dots mark outliers. The plotted results are the medians/means of all sampling locations from the dry plot as follows: Forest floor fluxes are determined as medians or means of measurements from three soil chambers (n = 3) with nine measurement repetitions per chamber, stem or shoot fluxes are expressed as medians or means of measurements on three trees (n = 3) with four to six repetitions per chamber. The fluxes from shoot and stem of each tree and from forest floor in its vicinity were always measured simultaneously thus allowing their comparison. Statistically significant differences at $p < 0.017$ (multiple comparison – Bonferroni correction) between flux components are indicated by different capital letters above bars.
